# Gossypiboma larynx: a rare cause of post-tracheostomy stridor—case report and review of literature

**DOI:** 10.1186/s13256-024-04490-7

**Published:** 2024-06-18

**Authors:** Gagan S. Prakash, Vikyath Satish, Bharath Raju, Neema Jayachamarajapura Onkaramurthy, Sathya Prakash

**Affiliations:** 1 Department of General Surgery, Bronx Care Health System, Bronx, NY USA; 2 Department of Pulmonology and Critical Care, Apollo Hospitals, 21/2 14th Main Jayanagar 1st Block, Bangalore, Karnataka 560011 India; 3 Department of Neurosurgery, Rutgers-Robert Wood Johnson University Hospital, New Brunswick, NJ USA; 4 Department of Internal Medicine, Columbia College of Physicians and Surgeons, NYC Health and Hospitals/Harlem Hospital Center, Harlem, NY USA; 5 Department of Thoracic Anesthesia, SDS and Rajiv Gandhi Institute of Chest Diseases, Bangalore, Karnataka India

**Keywords:** Bronchoscopy, Foreign body, Gossypiboma, Laryngeal membrane, Larynx, Stridor, Surgical gauze, Tracheostomy

## Abstract

**Background:**

Gossypiboma, a retained surgical sponge with a foreign body reaction, is an unusual but serious complication seen in open abdominal surgeries. It is exceptionally rare following head and neck surgeries. Here, we present a case of Gossypiboma of the upper airway following tracheostomy.

**Case presentation:**

A 32-year-old male presented with stridor and difficulty breathing one-month post-tracheostomy after a severe head injury following a road traffic accident. A neck radiograph was unremarkable, and a computed tomography (CT) scan of the neck showed a well-defined homogenous curvilinear membrane extending from the hypopharynx to the upper trachea. Bronchoscopic evaluation of the larynx and upper trachea revealed a retained surgical sponge, which was retrieved. The patient’s breathing improved drastically post intervention.

**Conclusion:**

Gossypiboma may go undetected in radiographs and may also present atypically as a homogenous membrane on a CT scan of the neck. Though rare, retained surgical items can have profound medicolegal and professional consequences on physicians. Hence, a strong clinical suspicion and vigilance for gossypiboma is necessary for patients presenting with respiratory distress post-tracheostomy.

## Background

Referred to euphemistically as Gossypiboma, the term denotes a mass lesion due to a retained surgical sponge surrounded by foreign-body reaction [1]. Retained sponges in adults occur most commonly in the abdomen (56%), pelvis (18%), and thorax (11%), but their incidence in head and neck surgeries/procedures is exceedingly rare [2], and clinicians face difficulty in early diagnosis. Failure in early diagnosis can lead to increased morbidity and mortality, given its potential to cause stridor and severe hypoxemia. We report a rare case of a retained surgical sponge in the larynx of a patient post-tracheostomy. To our knowledge, this is the first reported case of Gossypiboma in the upper airway.

## Case presentation

A 32-year-old male with no medical history who had undergone tracheostomy for a traumatic head injury one month ago returned to the hospital with complaints of exertional dyspnea and difficulty in breathing following tracheostomy decannulation. Physical examination revealed stridor, and differential causes like post-intubation/tracheostomy tracheal stenosis, pseudo-membrane, granulation tissue, and synechiae were suspected. The patient also complained of a foreign body sensation in the throat and an inability to expectorate secretions. He was clinically stable but had an elevated leukocyte count (13,400 cells/mm^3^) and was treated conservatively with steroids, antibiotics, and albuterol nebulization.

**Fig. 1:**
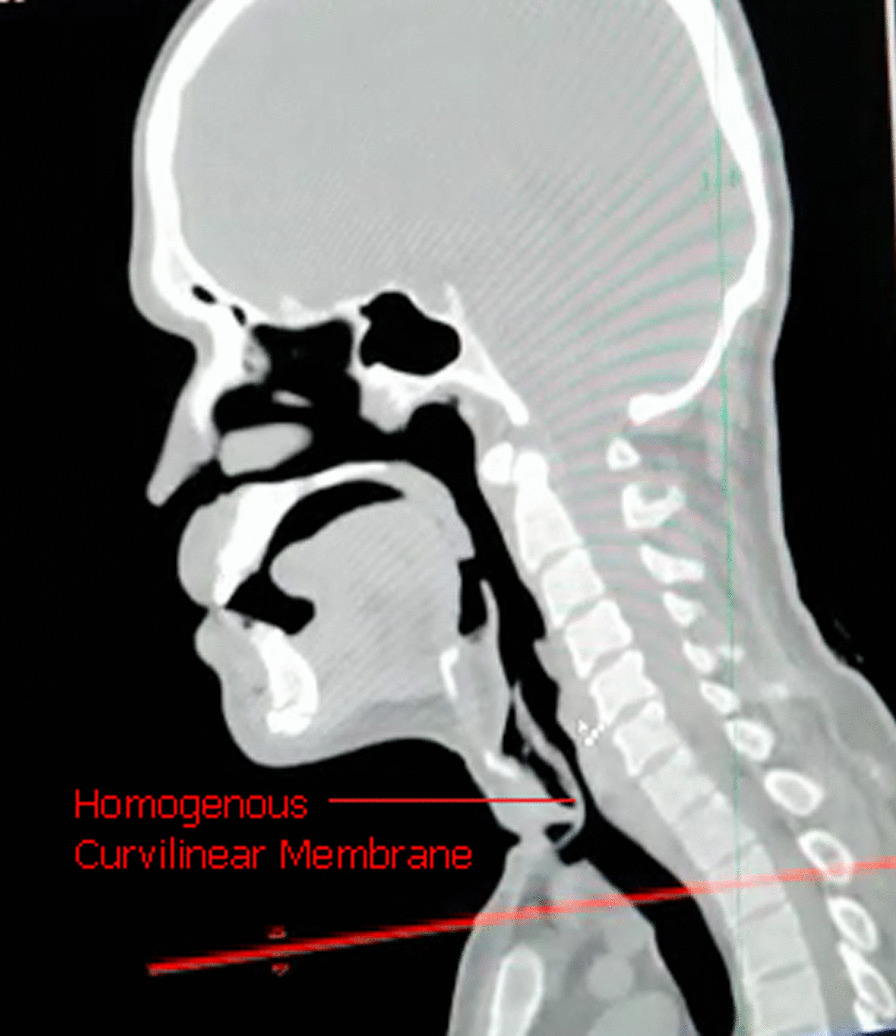
Homogenous curvilinear soft tissue density in the hypopharynx, larynx, and upper trachea causing mild to moderate luminal narrowing seen on CT scan of the neck

Since the chest radiograph was unremarkable, a CT scan of the neck was performed to delineate the pathology. It unveiled a well-defined homogenous curvilinear soft tissue density in the hypopharynx, larynx, and upper trachea (Fig. 1). Meanwhile, the patient developed an irritant cough and severe dyspnea at rest and underwent fiberoptic bronchoscopy for further management.

**Fig. 2:**
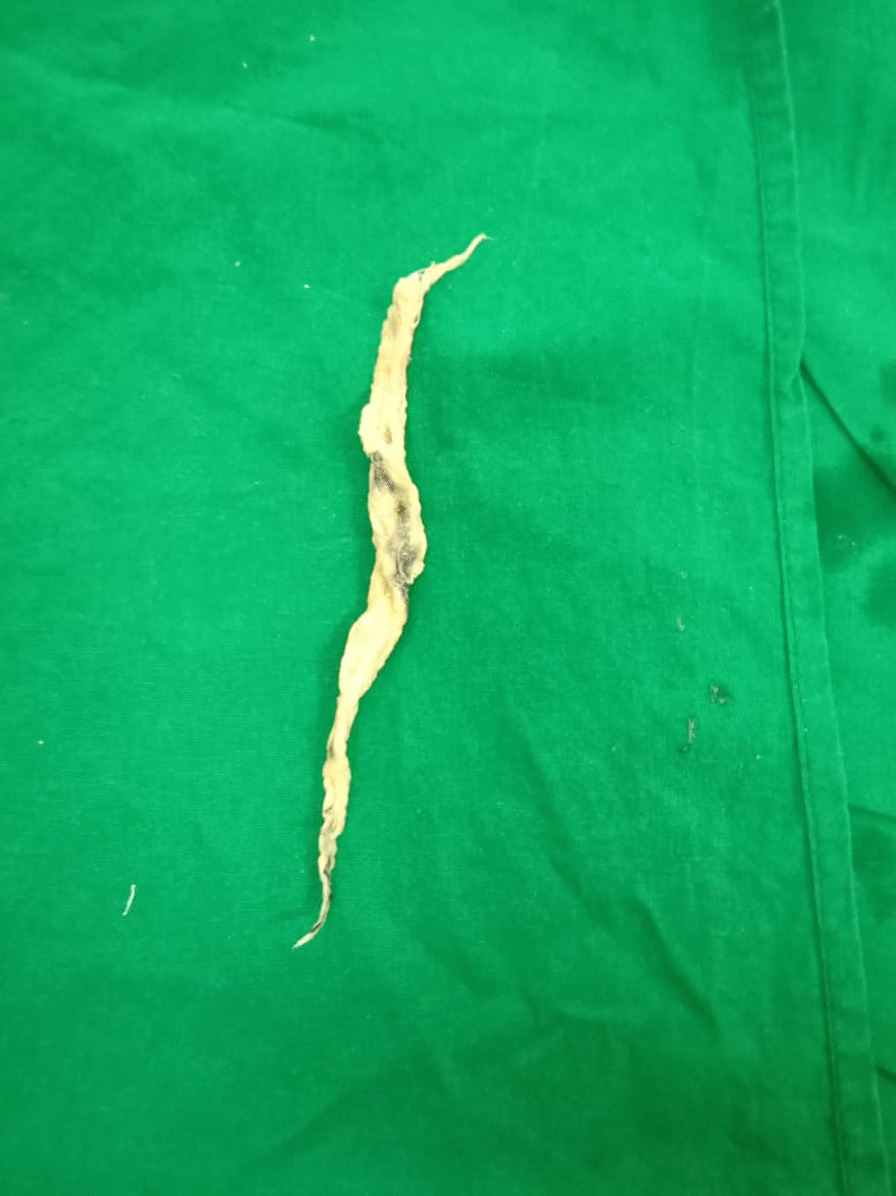
Gossypiboma retrieved from the larynx by bronchoscopy

During fiberoptic bronchoscopy, a greyish strand of glistening thick inspissated mass projecting across the vocal cords in the anterior commissure was observed. The scope was passed distally beyond the cords, and the band-like structure adhered to the anterior tracheal wall at the tracheostomy site. The cords appeared normal, with no granulation tissue or narrowing at the tracheostomy site. The tracheobronchial tree was inspected, and a thorough tracheobronchial toileting was performed. Upon withdrawal of the scope, the initial band-like structure in the larynx was not visualized anymore. Suspecting distal dislodgement, the entire tracheobronchial tree was re-visualized, revealing no band-like structure. Assuming it was a mucus strand that the patient coughed up and ingested, the bronchoscope was withdrawn. The patient complained of foreign body sensation in the throat during the visualization of the pharynx, where a thick greyish-black slimy mass was retrieved (Fig. 2). After thorough cleaning and examination, it was revealed to be a ribbon gauze piece covered with mucus. Following the procedure and removal of the gossypiboma, the patient improved clinically and was discharged the following day.

## Discussion

Gossypiboma is a well-accepted “never event” of the National Quality Forum, USA, and is also part of patient safety guidelines by the Health Department of the UK [3]. The incidence of Gossypiboma varies by surgical site and is exceedingly rare in the head and neck region. In 2005, the Joint Commission designated retained surgical items as a sentinel event requiring immediate root cause analysis investigation and response.

To our knowledge, this is the first reported case of a gossypiboma in the larynx. Most cases go unreported due to fear of litigation and are perceived to taint a professional career. The principle of “res ipsa loquitor” (let the act speak for itself) applies to such incidents and warrants a root cause analysis.

The most probable cause for the retained sponge was the surgical gauze used to guard the tracheostomy site. Typically, a gauze is tied around the tracheostomy tube for a good seal and to prevent air leaks. Here, the patient was shifted between multiple departments, which probably lead to the missing of the fact that the gauze was packed at the tracheostomy site in the first place. It is possible that the gauze got buried under the skin post-decannulation and slowly dislodged into the trachea over time. Despite the patient’s effort to cough it up, the gauze was lodged in the larynx as one end was embedded at the tracheotomy site. The gauze was dislodged and retrieved at the end of the bronchoscopy procedure. Gossypiboma larynx presents itself earlier than at other sites and is associated with an imminent risk of airway compromise, hence warranting early intervention.

Radiographs are the most commonly used modality to detect gossypibomas. In an unremarkable radiograph, imaging modalities like CT, Magnetic resonance imaging (MRI), and Positron emission tomography (PET) aid in the diagnosis.

Established methods to prevent retained surgical items during surgery, such as gauze counting, the pack of five approach, the Swiss cheese model, flattening the hierarchy of surgeons, and the use of a checklist have proven effective in lowering surgical mortality and litigation. However, owing to its exceedingly rare occurrence, sparse guidelines and studies exist on the prevention of gossypiboma in stomas. 

While the use of a radio-opaque marker-tagged surgical sponge [4] is universal in most Western surgical centers, its use is inconsistent in developing countries, especially outside major operating rooms. Surgical sponges tagged with a radiofrequency identification (RFID) chip and a handheld detector have a near-total detection rate, specificity, and sensitivity [5]. Lastly, effective communication and documentation between members of the multi-disciplinary [2] team can prevent the occurrence of a gossypiboma.

## Conclusion

Gossypiboma larynx is a rare albeit reversible cause of stridor in patients undergone tracheostomy. Gossypiboma may go undetected in radiographs and may also present atypically as a homogenous curvilinear membrane on a CT scan of the neck, an exceedingly rare finding. Early evaluation by bronchoscopy should be undertaken in the event of a diagnostic dilemma, which would aid in the definitive diagnosis and prove therapeutic. The primary goal is to establish hospital protocols for gossypiboma and use newer technology to avoid such “never events” from occurring.

## Data Availability

Not applicable.

## Abbreviations

CT: Computed tomography
MRI: Magnetic resonance imaging
PET: Positron emission tomography
RFID: Radiofrequency identification
